# Energetics of Paper Wasps (*Polistes* sp.) from Differing Climates during the Breeding Season

**DOI:** 10.3390/insects13090800

**Published:** 2022-09-01

**Authors:** Helmut Kovac, Helmut Käfer, Iacopo Petrocelli, Astrid B. Amstrup, Anton Stabentheiner

**Affiliations:** 1 Institute of Biology, University of Graz, 8010 Graz, Austria; 2 Dipartimento di Biologia, Università di Firenze, 50019 Sesto Fiorentino, Italy; 3 Department of Biology-Genetics, Ecology and Evolution, 8000 Aarhus, Denmark

**Keywords:** *Polistes* sp., energetics, temperature, climate

## Abstract

**Simple Summary:**

Paper wasps are widely distributed in nearly all regions of Europe. They are found in the warm Mediterranean, and in the harsh Alpine climate. Some species are very careful in the choice of nesting sites to ensure a proper development of the brood. We investigated microclimate conditions at the nests of three species (*P. dominula, P. gallicus, P. biglumis*) from differing climates, in order to characterize fine-scale environment conditions and conduct energetic calculations for an entire breeding season. The microclimate conditions (temperature) at the nests differed significantly in the Mediterranean, temperate, and Alpine habitats, but in all habitats the mean ambient nest temperatures were about 2 to 3 °C above the standard meteorological climate data. The wasps’ energetic expenditure depended strongly on temperature. *P. gallicus* from the warm Mediterranean climate exhibited the highest energetic costs, whereas *P. biglumis* from the harsh Alpine climate had the lowest costs during a breeding season. The energetic expenditure of *P. dominula* from the temperate climate was somewhat lower than in the Mediterranean species, but clearly higher than in the Alpine species. Temperature increase due to climate change may have a severe impact on the wasps’ survival as energetic costs increase.

**Abstract:**

Paper wasps are widely distributed in Europe. They live in the warm Mediterranean, and in the harsh Alpine climate. Some species are very careful in their choice of nesting sites to ensure a proper development of the brood. We investigated microclimate conditions at the nests of three species (*P. dominula, P. gallicus, P. biglumis*) from differing climates, in order to characterize environmental conditions and conduct energetic calculations for an entire breeding season. The mean ambient nest temperature differed significantly in the Mediterranean, temperate, and Alpine habitats, but in all habitats it was about 2 to 3 °C above the standard meteorological data. The energetic calculations of adult wasps’ standard and active metabolic rate, based on respiratory measurements, differed significantly, depending on the measured ambient temperatures or the wasps’ body temperatures. *P. gallicus* from the warm Mediterranean climate exhibited the highest energetic costs, whereas *P. biglumis* from the harsh Alpine climate had the lowest costs. Energetic costs of *P. dominula* from the temperate climate were somewhat lower than those in the Mediterranean species, but clearly higher than those in the Alpine species. Temperature increase due to climate change may have a severe impact on the wasps’ survival as energetic costs increase.

## 1. Introduction

Paper wasps of the genus *Polistes* are very successful with their primitively eusocial way of life, having a wide distribution and high abundances. Although the European species originate from the Mediterranean basin, they are now present in nearly all climatic regions of Europe. Climate and habitat conditions mainly determine the distribution ranges of the different species. They are found in natural landscapes and urban areas. The nesting habitat and nesting behavior differ between the species and depend on the local structures suitable for nesting sites and the microclimatic conditions (e.g., [1,2,3,4]). A generalist and highly successful species is *Polistes dominula*. It is a Palearctic species [1], which was originally common in the Mediterranean region, specifically the North African and Middle Eastern countries. In Europe, *P*. *dominula* is expanding its distribution range towards northern territories, and currently spreading in the northwest of Germany [5,6,7], the Netherlands [8], and the Baltic countries [9]. Some authors favor the nesting in sheltered places in human (including urban) environments as a reason for the establishment of *P. dominula* colonies in new regions [10,11,12,13], which enables range expansion from the favorable warm Mediterranean climate region into the temperate north of Europe. In contrast to *P. dominula*, *Polistes gallicus* is a common circum-Mediterranean species, highly abundant in its main distribution range, but still restricted to its original Mediterranean range. *P. gallicus* nests in natural landscapes and urban areas, but they are more abundant in less or non-urbanized areas [3,14]. A specialist concerning the habitat and climatic region is *Polistes biglumis*. *P. biglumis* is a boreo-montane species which mainly inhabits Alpine regions with harsher climatic conditions, e.g., in the Alps and in the Appennin. In Southern Europe, it inhabits mountain zones more than 1.500 m above sea level (1.600–2.350 m; [2]). The climate in their habitats greatly shortens the nesting season (four months duration on average) in comparison to species living at lower altitudes, e.g., *P. dominula* or *P. gallicus*. *P. biglumis* exhibits special adaptations in its nesting behavior due to the large diurnal temperature variations, choosing nesting sites which allow the use of solar radiation for nest thermoregulation [2,4].

In ectothermic insects, temperature is arguably the most important environmental variable driving the physiological rates and, therefore, is a key factor for survival and distribution. Polistine wasps are heterothermic insects, but they are ectothermic through most of their lifecycle [4,15,16,17]. How organisms respond to environmental temperature change will, among other parameters, determine species persistence in new environments. Ectothermic animals are considered particularly susceptible to environmental change since their body temperature and thus physiological performance vary acutely with environmental conditions. Consequently, changes in ambient temperature affect physiology, altering individual performance and fitness. Metabolic rate is a key component of energy budgets and a crucial parameter for the survival of insects in a changing environment. It is a fundamental physiological index of an organism’s energetic and material needs, its processing capacity and its ecological impact [18]. Metabolic rates of ectotherms depend principally on body mass and body temperature [18]. The metabolism of ectotherms is temperature-dependent, and the exponential nature of the metabolic rate– temperature relationship means that warmer temperatures are disproportionately affecting the energy balance. Metabolic rate is suggested to reflect the energetic costs of adaptation to a particular thermal environment [19,20] (see also [21]), rather than a purely mechanistic response to temperature. As a consequence, species living in different thermal habitats may have evolved different levels of metabolic control, suggesting different levels of vulnerability to warming (e.g., [21,22,23,24,25]). In an investigation on paper wasps from differing climates, Kovac et al. [17] demonstrated their differing sensitivity of the metabolism to temperature.

The need to identify the ecological consequences of climate change has boosted the development of macroecological approaches and climatological tools (e.g., Worldclim climate data provider, https://www.worldclim.org/, accessed on 26 August 2022) that can be used in predictive models to anticipate the consequences of climate warming and future changes in biological systems. However, for energetic calculations, global temperature models ignoring fine-scale microhabitat deviations are not adequate. For more accurate calculations, the actual microclimatic conditions experienced by organisms in their habitat are much more appropriate [26,27,28]. In order to characterize the nest microclimate from different habitats and climate regions, we investigated the environmental conditions at the nests of the three above-mentioned *Polistes* species. The goal of these measurements was to estimate the wasps’ energetic demand at the nest during an entire breeding season. Therefore, we measured the ambient air temperature and radiation at the nest and used these data for energetic calculations. The energetic calculations were conducted using temperature-dependent metabolic rate models of the three *Polistes* species, *P. dominula*, *P. gallicus* and *P. biglumis* [17,29], and adequate body temperature models [4,17]. The results revealed characterization of the differing microclimates of the three species’ habitats, and the consequences thereof for the energy budget of the wasps.

## 2. Materials and Methods

### 2.1. Species, Locations, and Climates

**Figure 1:**
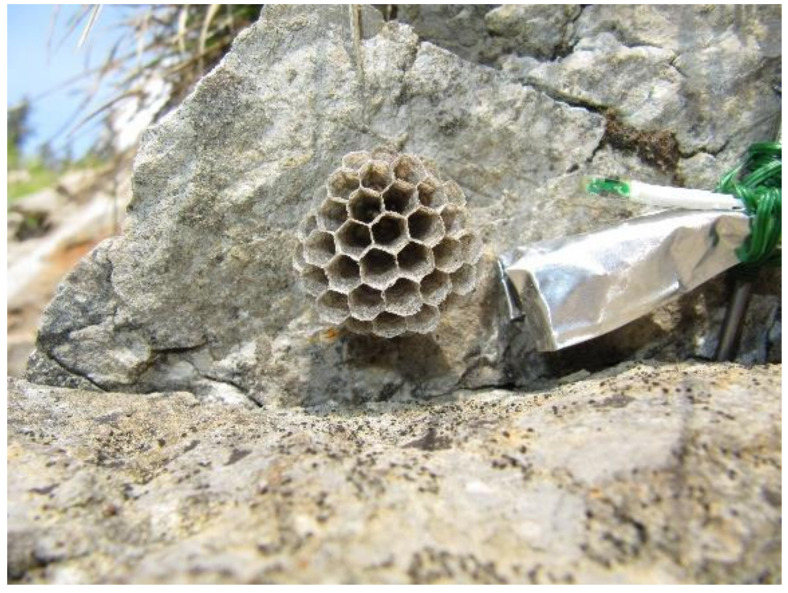
Nest of *Polistes biglumis* provided with a temperature and radiation sensor. The temperature sensor is protected from the solar radiation.

The investigations were conducted on nests of *Polistes dominula* (8 nests)*, Polistes gallicus* (6 nests), and *Polistes biglumis* (7 nests) in their natural habitats. The nests of *P. dominula* were located in lofts or bird boxes in rural areas of Styria (Austria) in the temperate climate of Central Europe. The nests of *P. gallicus* were found at sheltered sites at the face of different buildings and in grave lanterns at a cemetery in a predominantly rural area of Tuscany (Italy) in the Mediterranean climate of southern Europe (see also [4]). The nests of *P. biglumis* (Figure 1) were located in a mountainous landscape in the Alpine climate region of Carinthia and Styria (Austria). Their nests were built on rocks or stone walls.

**Figure 2:**
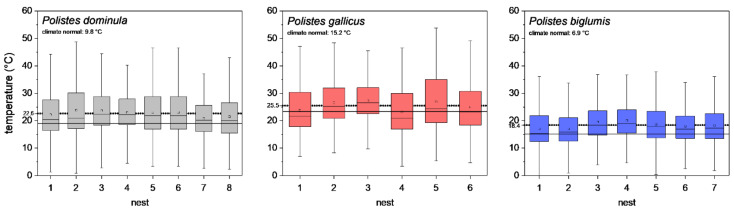
Ambient air temperatures at paper wasp nests during a breeding season (May–October 2018–2020). Box and whisker plots represent median temperatures with first and third quartiles, dots in plots are means. The dotted line indicates the mean of all nests, and the solid line shows the meteorological standard mean ambient temperature (May–October 2018–2020) of the nearest weather stations. The climate normal values (1981–2010) of the nearest weather stations (*P. dominula*: Graz, *P. gallicus*: Firenze, and *P. biglumis*: Kornat) are indicated. All single nest temperatures of *P. dominula*, *P. gallicus*, and *P. biglumis* differed significantly from the meteorological standard ambient temperature of the nearest weather stations (*p* < 0.05, pairwise Bonferroni test).

To describe the different climates of the three habitats, the climate normal values (1981–2010; [30,31]) of the nearest weather stations were evaluated, and are indicated in Figure 2. Furthermore, for comparison with our microclimate measurements, the mean temperature of the meteorological standard air temperature measurement (2 m height and shaded) of these weather stations were calculated for the breeding season (May–October 2018–2020).

### 2.2. Temperature and Radiation Measurement

The measurements were conducted during the wasps’ entire breeding season (May–October) in the years 2018–2020. Nest ambient temperature and radiation were recorded continuously with data loggers (MSR Electronics GmbH, Seuzach, Switzerland; and Extech SD 200, FLIR Commercial Systems, Nashua, NH, USA) in 10-min intervals in close vicinity to the nests (Figure 1). The temperature and radiation sensors were fixed 2–5 cm beside the nest. The temperature sensor was protected by an aluminum cover to avoid heating by direct solar radiation. As *P. dominula* nests were always shaded (global radiation < 100 W m^−2^), we measured only ambient temperature at these nests.

### 2.3. Energetic Expenditure Calculations

We used models describing the relationship between temperature and metabolic rate from previous studies ([17,29]; Table S1) in which respiration rates were measured in the three species we investigated in the present study. The carbon dioxide production for 10-min intervals of each nest was calculated from climate data measurements of this study using the equations of the standard metabolic rate (SMR: resting metabolic rate) and a mixed metabolic rate (MMR: mean of active metabolic rate and standard metabolic rate). The standard metabolic rate was evaluated in resting wasps and represents the metabolic costs of basal subsistence. The mixed metabolic rate is a combination of the SMR and active metabolic rate, representing mainly activities such as slow movements, grooming, and walking, similar to the main behavioral pattern at the nest (compare [32]). The metabolic costs of fanning, flight, and foraging were not included in this model.

First, we conducted the energetic calculations with the microclimate air temperature recordings from the nests (T_a_ model), assuming the wasps’ body temperatures were equivalent to the ambient temperature (according to the definition of “ectotherms”). To improve the model, we replaced the ambient temperature with the wasps’ body temperature (mean of head, thorax, abdomen; T_body_ model), calculated from models provided by Kovac et al. [17] and Stabentheiner et al. [4] (Table S2). Additionally, we conducted calculations with the mean temperature of the meteorological standard air temperature measurement (May–October 2018–2020, 2 m height and shaded) of the nearest weather stations (for *P. dominula* Graz: 9.8 °C, *P. gallicus* Firenze: 15.2 °C, *P. biglumis* Kornat: 6.9 °C), and with a 1 and 2 °C elevated ambient nest temperature, simulating assumed increased temperatures due to climate change.

The wasps’ energy turnover (J s^−1^ g^−1^) was calculated using the respiratory metabolic fit functions from previous studies ([17,29]). For this purpose, we first transformed the CO_2_ production to O_2_ consumption with the respiratory quotient determined for each species (RQ, *P. dominula*: 1.04, *P. gallicus*: 0.99, *P. biglumis*: 1.05; unpublished data) and then multiplied the O_2_ consumption with the adequate caloric equivalent of glucose (20.10 kJ L^−1^ O_2_). Then, the energetic turnover was calculated chronologically for the ten-minute intervals and summed for the entire breeding season. As the species differed significantly in weight, we used the mass specific metabolic rate for calculations and statistics. We have to emphasize that these calculations represent just the costs of nest activities of individual wasps during an entire breeding season (not foraging activity).

### 2.4. Data Analysis and Statistics

All calculations were performed with MS Excel (Microsoft Corporation, Redmond, WA, USA), and curve plotting was performed with Origin 2017 software (OriginLab Corporation, Northampton, MA, USA). The accompanying statistics were generated with Statgraphics software (Statgraphics Centurion XVI, StatPoint Technology Inc., The Plains, VA, USA) and IBM SPSS Statistics (SPSS Inc., Chicago, IL, USA). For the comparison between the climatic regions and the species, the nonparametric Kruskal–Wallis test was used.

## 3. Results

### 3.1. Nest Microclimate

The temperature measurements at the nests revealed a significant difference in the nest microclimate between the wasp species’ habitats during the breeding season (May–October). The mean ambient air temperature (±SD) at the eight *P. dominula* nests was 22.6 ± 8.07 °C. At the six *P. gallicus* nests, we measured 25.5 ± 8.56 °C on average, and at the seven nests of *P. biglumis*, mean temperature was 18.4 ± 6.83 °C (*p* < 0.001, Kruskal–Wallis test; all nests differed from each other; *p* < 0.05, pairwise Bonferroni test). The meteorological standard ambient temperature (May–October 2018–2020) recorded by the nearest weather stations was 19.0 ± 5.37 °C in Graz (*P. dominula*), 23.4 ± 5.67 °C in Firenze (*P. gallicus*), and 15.1 ± 5.25 °C in Kornat (*P. biglumis*) (*p* < 0.001, Kruskal–Wallis test; all weather stations differed from each other; *p* < 0.05, pairwise Bonferroni test). The nest ambient air temperatures in the three habitats were clearly elevated above the meteorological standard ambient temperature recorded by the nearest weather stations (temperature difference, *P. dominula*: 3.6 °C, *P. gallicus*: 2.4 °C, *P. biglumis*: 3.3 °C; Figure 2). All single nest temperatures of *P. dominula*, *P. gallicus*, and *P. biglumis* differed significantly from the meteorological standard ambient temperature of the nearest weather stations (*p* < 0.05, pairwise Bonferroni test).

### 3.2. Energetic Expenditure

**Figure 3:**
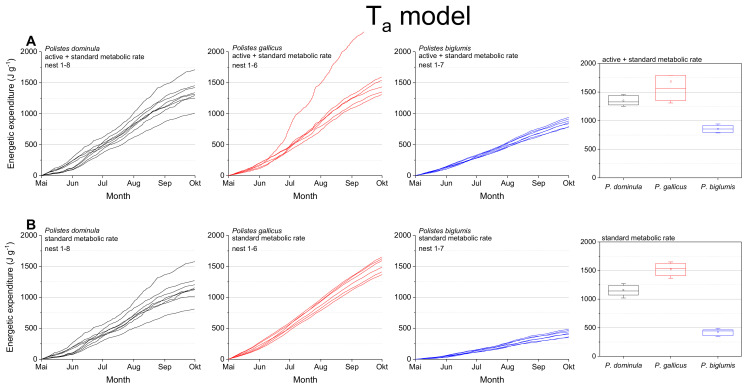
T_a_ model: Mass specific energetic expenditure of paper wasps during a breading season, calculated from ambient air temperature at the nest (T_a_) and metabolic rate-temperature models [17,29]. (**A**) Mixed activity (MMR, mean of active and standard metabolic rate), and (**B**) resting wasps (SMR, standard metabolic rate). Box and whisker plots represent median costs with first and third quartiles summed for the breeding season (May–October); dots in plots are means.

**Figure 4:**
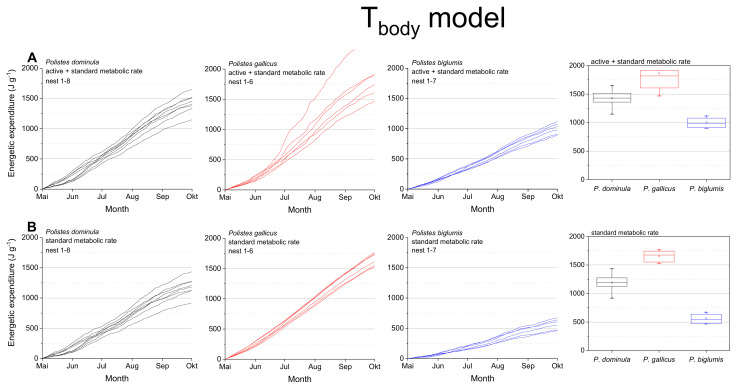
T_body_ model: Mass specific energetic expenditure of paper wasps during a breading season, calculated with models of the wasps’ body temperature (T_body_; [4]) and metabolic rate-temperature models [17,29]. (**A**) Mixed activity (MMR, mean of active and standard metabolic rate) and (**B**) resting wasps (SMR, standard metabolic rate). Box and whisker plots represent median costs with first and third quartiles summed for the breeding season (May-October); dots in plots are means.

The mass specific energetic expenditure was calculated for the standard metabolic rate (SMR) and the mixed metabolic rate (MMR: mean of active metabolic rate and standard metabolic rate) for the entire breeding season. Calculations were performed with ambient nest temperature as the independent variable (T_a_ model; Figure 3) and with the wasps’ body temperature (T_body_ model, Figure 4). The body temperature model used models describing the dependence of the wasps’ body temperature on ambient temperature and global (solar) radiation (Table S3 in Stabentheiner et al. [4]). The calculations using the ambient nest temperature as the main environmental parameter (T_a_ model; Figure 3, Table 1) revealed significant differences for the summed energetic values of the SMR and MMR between the species (in both SMR and MMR: *p* < 0.001, Kruskal–Wallis test; pairwise species comparison: *P. biglumis* differed from the other species but not *P. dominula* versus *P. gallicus*: *p* < 0.05, Bonferroni test). The energetic expenditure of the MMR was 1.12- to 2.03-fold higher than the SMR (Table 2).

The calculations using the body temperature as relevant parameter (T_body_ model; Figure 4, Table 1) also revealed significant differences for the summed energetic costs for the SMR and MMR between the species (in both SMR and MMR: *p* < 0.001, Kruskal–Wallis test; pairwise species comparison: *P. biglumis* differed from the other species but not *P. dominula* versus *P. gallicus*; *p* < 0.05, Bonferroni test). The energetic expenditure for the MMR was 1.20- to 1.78-fold higher than the SMR (Table 2). The energetic calculations using the body temperature showed higher costs than when using the ambient temperature (1.03- to 1.33-fold higher in the SMR, 1.06- to 1.17-fold higher in the MMR, Table 2).

The energetic calculations using the meteorological standard air temperature (May–October 2018–2020) yielded lower costs in all species (5.7 to 33.0% in the SMR, 18.9 to 31.1% in the MMR, Table 3), and the calculations with a 1 or 2 °C elevated temperature yielded higher costs in all species (4.5 to 24.7% in the SMR, 6.1 to 23.9% in the MMR, Table 3).

## 4. Discussion

We explored nesting habitats of paper wasps from three strongly differing climatic regions (Figure 2). Our investigations of the nest microclimate from these habitats and climates revealed significant differences in nest ambient air temperature during the breeding season. At the nests of *P. biglumis*, the species from the Alpine climate, we measured the lowest mean temperature and at the nests of *P. gallicus*, the species from the Mediterranean climate, the highest temperature (Figure 2). This was not a surprising result. However, a comparison of our own temperature measurements with standard meteorological data (May–October 2018–2020, standardized measurement at two meters height and shaded temperature sensors) of the nearest weather stations revealed interesting results. The mean ambient nest temperature in all habitats was clearly elevated above the standard air temperature data (2.4 to 3.6 °C; Figure 2). This is attained by the wasps’ selection of an appropriate nesting site in the habitat. Even in *P. biglumis*, in the harsh Alpine climate, we measured a 3.3 °C higher temperature at the nests. This is accomplished by a very careful selection of the nesting location. These wasps build their nests mainly oriented toward east-south-east to gain solar heat of the morning sun [2,4], similar to *Polistes nimpha* which also breeds in the open [33]. In *P. gallicus* from the warm Mediterranean climate, the difference to the standard meteorological data was the least, but nest temperature was nonetheless elevated by 2.4 °C. For *P. gallicus*, the main issue is overheating; therefore the species avoids nesting sites with direct insolation for the entire day to prevent the brood from unfavorable high temperatures. Our investigated population of *P. dominula*, nesting in the temperate climate of Central Europe, prefers sheltered places with a favorable, warm microclimate, which provides environmental conditions resembling that in the Mediterranean climate [17]. Their elevation above the standard meteorological temperature of 3.6 °C was the highest of the three species, which indicates the favorable microclimate at their nests.

In a convincing statement, Pincebourde and Salle [28] emphasized the importance of getting fine-scale temperature records near any surface of relevance for the investigated animal to explore their habitats’ environmental conditions. They state that the major pitfall in distribution modeling remains that the actual climatic conditions experienced by organisms in their microhabitat and across their home range are largely ignored [26]. The concept of “microclimate recordings” has to be applied to natural systems to detail the abiotic conditions experienced by organisms in their microhabitat. Such investigations are especially important in ectothermic insects, as their body temperature mostly corresponds to the ambient temperature. Only such microclimate and microhabitat data allow reliable energetic calculations and predictive distribution models. Significant deviations between macro- and microclimates are quite common in different ecosystems. Such deviations have been observed between leaf and air temperatures with consequences for leaf-dwelling organisms [34,35]. In this study, we also observed differences between macro- and microclimates. Our nest temperature measurements showed clear deviations from the standard climate recordings (Figure 2). These deviations towards higher temperature values may have a strong impact on the insect’s fitness and survival: first, they provide more favorable conditions for the brood development [4]; second, higher ambient temperatures imply a higher energetic expenditure for the basic subsistence (standard metabolic rate) and activity on the nest (active metabolism), which both increase exponentially with temperature in Polistine wasps [17,29,36]. Our data show clearly the dependence of the energetic expenditure on ambient temperature (Figure 3). In nests exposed to extremely high temperatures (Figure 3A; e.g., top nest in *P. gallicus*) the energetic expenditure is disproportionately enhanced due to the exponential increase of metabolism with temperature. In addition, the significantly lower costs of *P. biglumis* are caused both by the lower ambient temperature they were exposed to and by the overall lower metabolism compared with *P. dominula* [29]. The energetic calculations with the standard climate recordings clearly underestimated the energetic costs (by up to 33%, Table 3), thus emphasizing once more the need of fine-scale temperature records in the insects’ habitat.

The body temperature of ectotherms can deviate from ambient air temperature when the organism is exposed to the mosaic of microclimates at fine scales in their habitat (e.g., [4,37]). Radiation is a main source of body temperature increase in insects (e.g., [38,39]). In Polistine wasps it is especially important in the open-nesting species [4]. For this reason, we also conducted energetic calculations with the wasps’ mean body temperature in dependence on ambient temperature and global radiation when applicable [4,17] and got differing results (Figure 4). The calculated energetic expenditure was higher in all species, even in standard metabolic rate when the wasps were not active. This was especially pronounced in the Alpine species, *P. biglumis*, probably caused by the increased body temperature due to intense solar radiation [4]. Nevertheless, the energetic expenditure of *P. biglumis* was for both types of model calculation, with ambient temperature and body temperature as independent variables, significantly lower than in the other species. A main source of this difference is their generally lower sensitivity of metabolism to temperature variation [29]. Kovac et al. [29] suggested the harsh environmental conditions in the Alpine climate to force these wasps into an energy saving life style. The lower temperatures and more frequent bad weather conditions do not allow for a foraging frequency similar to that in the warmer lowlands. However, we have to emphasize that our calculations take into account only the costs at the nests and do not include costs of foraging flights or fanning for nest cooling. As flight is much more energy demanding [40], this should not be neglected in future total energetic expenditure estimations. However, observations on foraging time and behavior would be necessary.

Energy supply is essential for the insects’ survival in a world of global warming. To simulate energetic costs under future climate conditions, we also conducted model calculations with the simple assumption of an elevated ambient air temperature at the nests of 1 or 2 °C. With this assumption the energetic costs of mixed activities (MMR) increased by 10.4 or 20.0% in *P. dominula*, by 11.1 or 23.9% in *P. gallicus* and 6.1 or 12.4% in *P. biglumis* (Table 3). The impact on the energetic expenditure is higher in the species living in the warmer habitats, mainly due to the steeper exponential increase in metabolic rate with temperature. The species in the cooler habitat (*P. biglumis*) will also have higher costs, but they could also profit from an increased temperature. The foraging intensity (and efficiency) may increase with temperature, and the development time of the brood may be reduced. In addition, the possibility of longer periods of foraging may be beneficial for the food supply of the brood (with the assumption that enough prey is available), which could outweigh the higher costs due to higher temperatures. Global warming does not necessarily mean that temperature around the nests will always increase: possibly, geographic ranges of the wasps will move to the North, allowing insects to keep the current temperature conditions. However, it is not clear whether habitats in the North are always appropriate. The availability of sheltered nesting sites and the appropriate prey may be a limiting factor for the wasps’ distribution. Additionally, the overwintering may be a problem for the insects in colder regions, especially in the Mediterranean species *P. gallicus*.

## 5. Conclusions

Our investigation revealed the crucial importance of temperature on the energetics of ectotherms and the need of fine-scale microclimatic measurements in their habitat. The macroclimatic differences between the habitats of paper wasps are (partly) compensated for by the choice of an appropriate nesting site. A temperature increase due to climate change may have a strong impact on the insects’ energetics and survival, as higher temperatures mean higher costs for basic subsistence and activity.

## Figures and Tables

**Table 1:** Mass specific (summed) energetic expenditure (J g^−1^) of paper wasps of a breeding season (May–October), for resting wasps (SMR, standard metabolic rate) and mixed activity (MMR, mean of active and standard metabolic rate). Calculations with ambient air nest temperature (T_a_) and the wasps’ body temperature (T_body_) represent means and SD. Calculations with T_body_ consider the mean effect of T_a_ and Radiation on T_body_ according to Stabentheiner et al. [4]. N = number of nests.

Calculation with:	T_a_		T_body_		
Species	SMR	MMR	SMR	MMR	N
*P. dominula*	1161.2 ± 217.34	1350.0 ± 198.37	1193.5 ± 149.11	1426.4 ± 148.30	8
*P. gallicus*	1521.9 ± 118.13	1702.5 ± 440.36	1656.9 ± 104.59	1866.0 ± 383.38	6
*P. biglumis*	422.9 ± 50.38	858.5 ± 59.00	563.6 ± 79.87	1001.7 ± 81.17	7

**Table 2:** Relations between energetic expenditure of mixed activity (MMR = mean of active and standard metabolic rate) and standard metabolic rate (SMR), calculated with ambient air temperature (T_a_) and wasp body temperature (T_body_); see Table 1.

Calculation with:	T_a_	T_body_	T_body_ vs. T_a_	T_body_ vs. T_a_
Species	MMR/SMR	MMR/SMR	SMR/SMR	MMR/MMR
*P. dominula*	1.16	1.20	1.03	1.06
*P. gallicus*	1.12	1.13	1.09	1.10
*P. biglumis*	2.03	1.78	1.33	1.17

**Table 3:** Deviation of energetic costs of paper wasps during a breading season between different climate scenarios, calculated as difference between microclimate measurements and standard meteorological temperature data 2018–2020 (T_a_ (standard met.)) and future climate scenarios with a temperature increase of 1 °C or 2 °C above ambient temperature at the nest (T_a_ (future)). T_a_ (standard met.) temperature deviation from microclimate ambient nest temperature was −3.6 °C in *P. dominula*, −2.4 °C in *P. gallicus*, and −3.3 °C in *P. biglumis*). MMR = mixed activity metabolic rate, SMR = standard metabolic rate.

	MMR Difference to Microclimate (%)			SMR Difference to Microclimate (%)		
	***P. dominula***	***P. gallicus***	***P. biglumis***	***P. dominula***	***P. gallicus***	***P. biglumis***
T_a_ (standard met.)	−31.1	−21.0	−18.9	−33.0	−5.7	−32.1
T_a_ (future +1 °C)	10.4	11.1	6.1	11.7	4.5	9.1
T_a_ (future +2 °C)	20.0	23.9	12.4	24.7	9.0	20.4

## Data Availability

All data are available in the paper and the Supplementary Information.

## Supplementary Materials

The following supporting information can be downloaded at: https://www.mdpi.com/article/10.3390/insects13090800/s1, Table S1: Functions and parameters; Table S2: Functions and parameters.
