# Supplementary material for: Energetics of Paper Wasps (Polistes sp.) from Differing Climates during the Breeding Season
Source: Insects. 2022 Sep 1;13(9):800. doi: 10.3390/insects13090800 (PMC9501522; doi:10.3390/insects13090800)
Supplement: Supplementary file 1 [file insects-13-00800-s001.zip › insects-1853712-supplementary.pdf]

insects

Energetics of paper wasps (*Polistes* sp.) from differing climates during the breeding season.

Helmut Kovac[1*], Helmut Käfer[1], Iacopo Petrocelli[2], Astrid B. Amstrup[1,3], Anton Stabentheiner[1*]

[1] Institute of Biology, University of Graz, 8010 Graz, Austria
[2] Dipartimento di Biologia, Università di Firenze, 50019 Sesto Fiorentino, Italy
[3] Department of Biology - Genetics, Ecology and Evolution, 8000 Aarhus C, Denmark
* Correspondence: helmut.kovac@uni-graz.at; anton.stabentheiner@uni-graz.at

Supplementary Information

Table S1. Functions and parameters for calculation of the wasps' mixed metabolic rate (MMR) and standard metabolic rate (SMR) according Kovac et al. (2017) and Kovac et al. (2020). In *P. gallicus* the SMR was calculated with a linear function, all other MMR and SMR with exponential functions. T_a = ambient air temperature.

Species/Function	Parameter	MMR	SMR
P. dominula			
$y = y0 + A*exp(R0*T_a)$	y0	-65.626	-3.10356
	A	46.443	15.36177
	R0	0.08296	0.10942
P. gallicus			
$y = y0 + A*exp(R0*T_a)$	y0	104.58839	-50.69017
$Y = y0+A*T_a$	A	3.19731	15.39015
	R0	0.14276	
P. biglumis			
$y = y0 + A*exp(R0*T_a)$	y0	-149.2341	-65.42187
	A	181.74669	46.57254
	R0	0.03462	0.06316

Table S2. Functions and parameters for calculations of the wasps' body temperature according Kovac et al. (2017) and Stabentheiner et al. (2022). T_a = ambient air temperature.

Species / Function	Parameter	Value
P. dominula		
$T_{body} = a*(1 + (d-1)*exp(-k*(T_a-xc)))^(1/(1-d))$	a	39.36392
	xc	30.11685
	d	10.67527
	k	0.36288
P. gallicus		
$T_{body} = a + xc*T_a + d*Radiation$	a	4.49252
	xc	0.893964
	d	0.00337853
P. biglumis		
$T_{body} = a + xc*T_a + d*Radiation$	a	0.540869
	xc	1.0583
	d	0.00652941
